# New pharmacological agents and emerging therapeutic targets for painful diabetic neuropathy

**DOI:** 10.3389/fendo.2026.1788702

**Published:** 2026-03-31

**Authors:** Theodoros Panou, Nikolaos Papanas, Peter Kempler

**Affiliations:** 1 Diabetes Centre-Diabetic Foot Clinic, Second Department of Internal Medicine, Democritus University of Thrace, Alexandroupolis, Greece; 2 Semmelweis University, Department of Medicine and Oncology, Budapest, Hungary

**Keywords:** diabetic neuropathy, neuropathic pain, painful diabetic peripheral neuropathy, treatment, type 1 diabetes mellitus, type 2 diabetes mellitus

## Abstract

Painful diabetic peripheral neuropathy (PDPN) remains a serious complication of diabetes mellitus (DM). Recent clinical trials have demonstrated promising outcomes for pilavapadin (LX9211), an orally administered selective, potent inhibitor of adapter protein-2-associated kinase 1 (AAK1) and vixotrigine, a broad spectrum voltage-gated sodium channels (Navs) inhibitor. Their beneficial role was reflected in improved average daily pain (ADP) score and Patient Global Impression of Change (PGIC). Gamma-aminobutyric acid (GABA) receptor agonism has yielded favourable outcomes in preliminary studies. Experimental studies have further expanded the spectrum of potential agents, therapeutic targets and mechanisms in PDPN. Some of potential therapeutic approaches include chemokine suppression (CCR2/CCR5 or CXCR1/2 inhibition) and transient receptor potential vanilloid 1 (TRPV1) pathway suppression. Impaired mitochondrial function in PDPN is now being discussed and the inhibition of poly (adenosine diphosphate [ADP]-ribose) polymerase 1(PARP1), a mitochondrial enzyme responsible for deoxyribonucleic acid (DNA) repair is emerging. Local treatments have also been examined in animal models, such as resiniferatoxin. Limited evidence exists regarding the therapeutic potential of antidiabetic agents, glucagon-like peptide-1 receptor agonists (GLP-1RAs), being most widely studied in experimental settings. Future large clinical trials are now required to confirm the efficacy of novel promising agents and to delineate further potential favourable effects of antidiabetic agents in clinical settings.

## Introduction

Diabetic peripheral neuropathy (DPN) pertains mainly to sensory function deficiencies which culminate progressively and are accompanied by severe pain and enhanced morbidity (1). This length-dependent peripheral nerve complication occurs almost in 1 out of 2 subjects with type 2 diabetes mellitus (T2DM) and type 1 diabetes mellitus (T1DM) during their lifetime, rendering DPN the most frequent complication of diabetes mellitus (DM) (2).

Painful diabetic peripheral neuropathy (PDPN) affects almost 20% to 30% of subjects with T2DM or T1DM (3). The pooled global prevalence of PDPN was 46.7% in a recent meta-analysis (4). In a nationwide cross-sectional study from China, prevalence was even higher, reaching 57.3% (5). The EURODIAB prospective study identified female sex as a major risk factor for PDPN development (6). To date, emphasis is placed on on use of gabapentinoids, serotonin-norepinephrine reuptake inhibitors, tricyclic antidepressants, and sodium channel blockers for pain alleviation in PDPN (7, 8). In addition, physicians are encouraged to address autonomic neuropathy, thereby ensuring comprehensive symptom relief (7). Due to suboptimal effects of current standard-of-care for PDPN management, combinations of these medications are frequently utilised in clinical practice (9, 10). Non-pharmacological therapies have been introduced as well, e.g. high-frequency spinal cord stimulation (11). This variety notwisthstanding, treatment often fails, and multiple therapeutic shifts are commonly required to achieve pain relief (12–14).

Current research focuses on identifying molecular targets and on developing novel causative therapeutic agents for the management of PDPN beyond symptomatic pain relief. In this context, the aim of the present narrative review was to explore novel treatment strategies and therapeutic targets for the management of PDPN.

## Search strategy

An electronic search in Scopus, PubMed/MEDLINE, and Google Scholar for published articles from 01.01.2022 to date was carried out, using combinations of the following key words: “diabetic neuropathy”, “painful diabetic peripheral neuropathy”, “painful diabetic neuropathy”, “diabetes mellitus”, “treatment”, “diabetic neuropathic pain” and “neuropathic pain”. All types of articles (clinical trials, meta-analyses, case-control studies, observational studies, cross-sectional studies, prospective/retrospective studies, cohort studies, comparative studies, randomised studies, experimental studies) were included. Only articles in English were considered.

## Clinical studies

### The promising perspective of targeting sigma-1 receptors and gamma-aminobutyric acid

Targeting sigma-1 receptor (S1R) has been investigated as a potential way of mitigating PDPN through the use of E-52862 (15). Targeting S1R has been previously evaluated as a therapeutic approach in several pain-associated conditions, such as herpes zoster, acute post-operative pain and chemotherapy-induced neuropathy (16). S1R, as an endoplasmic-reticulum-resident transmembrane protein is not similar to other opoiod receptors (μ-, κ-, and δ-), which are G protein-coupled receptors (GPCRs) (16, 17). Recent insights in the molecular structure of the receptor and its ligands have attracted interest (17, 18).

A phase 2, randomised, double blind, placebo-controlled, proof-of-concept study evaluated 279 subjects, either with chronic post-operative pain or PDPN (15). Overall 163 subjects with PDPN were included and 85 participants received E-52862 (15). Subjects with both DM types were included: 12 subjects with T1DM and 73 subjects with T2DM in the treatment arm and 11 subjects with T1DM and 67 subjects with T2DM in the control arm (15). Efficacy was seen in subjects with chronic post-operative pain (least squares mean difference [LSMD]: -0.9, p=0.029). By contrast, the effect of E-52862 on PDPN has not been similarly encouraging (15). At baseline, similar average pain scores were seen in the intervention and the control group, according to numerical pain rating scale (NPRS) (15). NPRS allows participants to evaluate their pain perception on a scale from 0 to 10 and is regarded a traditional approach for pain evaluation in clinical studies (19). DPN was assessed by Michigan Neuropathy Screening Instrument (MNSI), a validated assessment tool for DPN (15, 20). An insignificant reduction in average pain was seen in subjects treated with E-52862 compared with those on placebo (-0.1, 95% confidence interval [CI]: -0.6 to 0.4, p=0.766) (15). In respect to worst pain reduction, similar decreases were observed in intervention and control group (p=0.461) (15). The difference between E-52862 and placebo in 30% pain reduction (62.4% vs. 59.0% for ≥30% reduction, p=0.856) and in 50% pain reduction (38.8% vs. 34.6% for ≥50% reduction, p=0.636) was not significant (15). A subanalysis on Brief Pain Inventory-Short Form (SF-BPI) items 5 (pain on average) and 3 (pain at its worst) showed no superiority of E-52862 vs. placebo in pain severity (p=0.478) and pain interference (p=0.112) (15) (**Table 1**).

**Table 1:** Clinical studies on novel therapeutic agents in PDPN.

Study	Study type	Study design	Main outcomes	Conclusion
Gálvez et al. (2025) (15)	Phase 2, randomised, double blind, placebo- controlled, proof- of-concept study	163 subjects with PDPN -85 subjects with PDN treated with E-52862 with mean age 61.0 years, mean HbA_1c_ 7.6%, mean DM and PDPN duration 13.0 and 2.3 years and mean MNSI 5.1 (12 subjects with T1DM and 73 subjects with T2DM) -78 subjects with PDN treated with placebo with mean age 60.5 years, mean HbA_1c_ 7.3%, mean DM and PDPN duration 10.3 and 2.2 years and mean MNSI 5.0 (11 subjects with T1DM and 67 subjects with T2DM)	**Efficacy**: -Mean baseline average pain score was 5.3 for subjects on E- 52862 vs. 5.4 for subjects on placebo -Change from baseline for average pain was -2.2 on E-52862 vs. -2.1 on placebo, (p=0.766) - Mean baseline worst pain score was 6.6 on E- 52862 vs. 7.1 on placebo -Change from baseline for average pain was -2.4 on E-52862 vs. -2.6 on placebo (p=0.461) **Safety**: -Mild dizziness, nausea, headache, and anxiety were the most frequent adverse effects, without being associated with E-52862 **Limitations**: -Increased response in the control arm -Limited study duration -Restricted number of study participants, mostly white	-Further exploration of S1R antagonist in PDN is encouraged
Guo et al. (2024) (21)	Phase 2 to 3 adaptive, multi- centre, double blind, placebo- and pregabalin- controlled randomised clinical trial	725 subjects with PDPN with mean age 58.8 years and with mean BMI 24.49 kg/m^2^ -178 subjects treated with 40 mg HSK16149 -180 subjects treated with 80 mg HSK16149 -66 subjects treated with 120 mg HSK16149 -64 subjects treated with 160 mg HSK16149 -177 subjects treated with placebo -62 subjects treated with 300 mg pregabalin 363 subjects treated with 40, 80, 120 or 160 mg HSK16149, 300 mg pregabalin or placebo in Stage 1 362 subjects with PDPN treated with 40 or 80 mg HSK16149 or placebo in Stage 2	**Efficacy**: -In the full-set analysis at week 5: mean reduction in ADPS was 1.12 in the 40 mg group, 1.13 in the 80 mg group, 1.03 in the 120 mg group, 1.13 in the 160 mg group for subjects on HSK16149, 0.75 in the control group and 1.02 in the pregabalin group -In stage 2 at week 5: mean reduction in ADPS was 2.24 in the 40 mg group, 2.16 in the 80 mg group, 2.03 in the 120 mg group, 2.15 in the 160 mg group for subjects on HSK16149, 1.23 in the control group and 2.09 in the pregabalin group **Safety**: -Adverse events were reported by 75.1%; mild-to- moderate dizziness and somnolence were the commonest **Limitation**: -Adaptive study design	-HSK16149 holds therapeutic potential for DPNP
Pop-Busui et al. (2024) (24)	Double-blind, randomised, placebo- controlled, proof- of-concept study	319 subjects with PDPN with mean age 62.0 years, mean HbA_1c_ 7.7%, with mean BMI 32.1 kg/m^2^ and with mean PDPN duration 6.6 years (15 subjects with T1DM, 257 subjects with T2DM and 47 subjects without particular DM type reported): -106 subjects treated with 10 mg LX9211 -106 subjects treated with 20 mg LX9211 -107 subjects in the control arm	**Efficacy**: -Treatment with 10 mg LX9211 significantly reduced ADPS by 1.39 vs. 0.72 in the control arm at week 6 (mean difference: 0.67, 95% CI: -1.16 to -0.18, p=0.007) - Treatment with 20 mg LX9211 significantly reduced ADPS by 1.27 vs. 0.72 in the control arm (mean difference: -0.55, 95% CI: -1.06 to -0.05, p=0.030) -Significance was observed at weeks 1,2, 3 and 4 for both doses vs. placebo and at week 5 for the low dose (p<0.05) -At week 6, improved NPSI score (p=0.008 for low-dose and, p = 0.064 for high-dose) and burning pain (p=0.008 for low-dose and, p=0.064 for the high-dose) was found -Self-perceived health status improvement (according to PGIC) was significantly more frequent in low-dose group vs. controls (mean: -0.35 95% CI: -0.67 to -0.03, p=0.031), but not in high- dose group (mean: -0.15, 95% CI: - 0.48 o- 0.17, p=0.351) **Safety**: -Common adverse effects of mild-to-moderate intensity included dizziness, nausea, headache, constipation, balance disorder, somnolence, and vomiting -Most cases of treatment discontinuation were reported within the 2 first weeks (in the low-dose LX9211 group) and within the 6 weeks (in the high-dose LX9211 group), mostly attributed to dizziness, headache, nausea, and fatigue -Similar incidence of adverse events in all study arms: (21.2% in the low-dose LX9211 group, 19.6% in the high-dose LX9211 group and 14.4% in the control arm) **Limitations**: -Short study duration -Availability of evidence exclusively from participants in the United States	-LX9211 is beneficial for PDPN
Hong et al. (2024) (29)	Model-based meta-analysis	Clinical trials involving pregabalin and mirogabalin for DPNP and post- herpetic pain (exclusively pregabalin)	**Efficacy**: -ADPS was reduced by 8.86 % (95 %: CI:4.6- 12.94%) on pregabalin and 7.57 % (95 % CI: 1.1-14.3 %) on mirogabalin **Safety**: -Dizziness, somnolence, peripheral oedema, weight increase, and headache were the commonest adverse events; with the exception of the latter, adverse events were more frequent vs. controls -For mirogabalin users, RR for dizziness, somnolence, oedema peripheral, and weight gain were 3.59 (95 % CI: 2.31-5.60), 3.74 (95 % CI: 2.57- 5.43), 4.88 (95 % CI: 2.35-10.11), and 7.12 (95 % CI: 2.78-18.25), respectively -Dropout rates: 12.1% on mirogabalin vs. 17% on pregabalin and 15.1% in the control arm **Limitations**: -Exclusive comparison of microgabalin with pregabalin	-Microgabalin is not superior in PDPN and post-herpetic pain vs. pregabalin
Michelson et al. (2023) (30)	Phase 2, 12- week, randomised, double-blind, placebo- controlled study	282 subjects with painful PDPN: -142 subjects treated with ricolinostat with mean age of 59.5 years, mean HbA_1c_ 7.9%, with mean BMI 31.3 kg/m^2^ and with mean DM duration 14.2 years and mean PDPN duration 6.6 years -140 subjects in the control arm with mean age of 60.9 years, mean HbA_1c_ 7.8%, with mean BMI 32.2 kg/m^2^ and mean DM duration 13.6 years and mean with mean PDPN duration of 6.1 years	**Efficacy**: -Reductions in NRS were 1.2 on ricolinostat and 1.0 in the control arm (p=0.38) -Reductions in UENS were 1.51 on ricolinostat and 1.80 in the control arm (p=0.56) -Reductions in BPI-SF were 0.95 on ricolinostat and 1.03 in the control arm (p=0.74) -Reductions in NTSS-6 were 2.1 on ricolinostat and 2.3 in the control arm (p=0.72) -Reductions in Norfolk QOL- DN were 7.6 on ricolinostat and 7.8 in the control arm (p=0.93) -Identical improvement in PGIC 6 for subjects on ricolinostat and for subjects in the control arm -At week 24: reduction in NRS by 1.9 on ricolinostat and by 1.7 in the control arm; reduction in UENS by 1.5 for subjects on ricolinostat and by 2.2 for subjects in the control arm **Safety**: -Urinary tract infections (3.5%), nausea (2.1%) and diarrhoea (2.1%) were the 3 most common adverse events -27% of subjects on ricolinostat and 19% of subjects in the control arm experienced a drop of neutrophils by >500 X 10^3^/mm^3^ **Limitations**: -Short study duration -Sensory phenotypes potentially responsive to HDAC6 inhibition not identified	-No promising effects of ricolinostat for PDPN
Faber et al. (2023) (34)	Phase 2, multi- centre, placebo- controlled, double-blind, enriched-enrolment, randomised withdrawal study (CONVEY)	-265 subjects with SFN -234 subjects in the open-label study (all participants on 350 mg) -123 subjects in the double-blind study (73.8% of subjects with DM-associated SFN and 26.2% of subjects with idiopathic SFN) -40 subjects on 200 mg vixotrigine with SFN duration 74.3 months -41 subjects on 350 mg vixotrigine with SFN duration of 60.6 months -41 subjects in the control arm with SFN duration 59.8 months	**Efficacy**: -In the open- label study, ADPS ranged between +1.8 and -7.7 -A significant decrease in ADPS was found only in the group treated with 200 mg vs. the control arm (mean difference: -0.85, 95% CI: -1.71 to 0.00, p=0.050), without significant effect in the group treated with 350 mg (mean difference: -0.17, 95% CI; -1.01 to 0.68, p=0.70) from baseline to week 12 - A significant decrease in ADPS was found only in the group treated with 200 mg vs. control arm (mean difference: -0.85, 95% CI: -1.74 to 0.03, p=0.058), without significant effect in the group treated with 350 mg (mean difference: -0.32, 95% CI: -1.19 to 0.55, p=0.47) from randomisation to week 12 -Among subjects with DM- associated SFN: a beneficial effect on 200 mg vs. controls (mean difference: -0.94, 95% CI: -1.85 to -0.03), but not on 350 mg (mean difference: -0.25, 95% CI: -1.18 to 0.69) -At week 12: significant reduction in WDPS on 200mg vs. controls group (mean difference: -0.93, 95% CI: -1.85 to -0.02, p=0.046, but not on 350 mg (mean difference: -0.25, 95% CI: -1.15 to 0.65, p=0.58) - Significant improvement in PGIC on 350 mg vs. controls (48.8% vs. 30.0%, OR: 2.60, 95% CI: 0.97-6.99, p=0.058), but not on 200 mg -No significant improvement in sleep interference score (200 mg: - 0.47, 95% CI: -1.30 to 0.36; 350mg: -0.08, 95% CI: -0.89 to 0.74), NPSI total score (200 mg: - 2.9, 95% CI: -13.3 to 7.6; 350mg: 1.30, 95% CI: -11.20 to 8.50), NPSI sum score of burning and pressing (200 mg: -0.47, 95% CI: -2.79 to 1.84; 350 mg: 0.07, 95% CI: - 2.11 to 2.26) or mean BPI-SF interference score (200 mg: 0.60, 95% CI: -1.65 to 2.84; 350mg: -0.65, 95% CI: -2.52 to 1.22) **Safety**: -In the open- label study, headache and dizziness of mild-to-moderate severity were the most frequent events (in 9.4% of participants each) -In the double- blind study, falls, nasopharyngitis, muscle spasm and UTI were mostly reported **Limitations**: -Early study termination -Short study duration -Insufficient (mostly White) and clinically heterogeneous sample	-Vixotrigine is a promising agent for PDPN
Tiecke et al. (2022) (36)	Phase 2a, randomised, dose-finding, proof of concept study	88 subjects with PDPN with mean age of 67.0 years, with mean BMI of 29.9 kg/m^2^ time since diagnosis of DM 15.3 years with mean time since diagnosis of neuropathy 3.9 years (91.9% of study participants with T2DM) -22 subjects treated with 10 mg NRD.E1 -22 subjects treated with 40 mg NRD.E1 -21 subjects treated with 150 mg NRD.E1 -21 subjects in the control arm	**Efficacy**: -Decreased mean of daily average NRS pain intensity on 10 mg (placebo- corrected treatment effect: 0.42, 95% CI: -1.50 to 0.66; p=0.438), on 40mg (placebo- corrected treatment effect: 0.82, 95% CI: 0.07 to 1.58, p=0.034) and on 150 mg (placebo- corrected treatment effect: 0.66, 95% CI: -0.03 to 1.35; p=0.061) -Placebo- corrected NRS reductions: 0.52 (95% CI: -1.76 to 0.71) on 10 mg, 1.46 (95% CI: 0.26 to 2.66) on 40 mg and 1.20 (95% CI: 0.10 to 2.29) on 150 mg -Standardised effect size: -0.67 (95% CI: -1.29 to -0.05) on 40 mg and -0.60 (95% CI: -1.22 to 0.03) on 150 mg -In the sub- population of modified intent- to-treat: subjects who had confirmed moderate or severe pain showed placebo- corrected reductions by 1.69 (95% CI: - 3.29 to -0.09) for 10 mg, by 2.66 (95% CI: -4.15 on -1.18) on 40 mg and by 1.78 (95% CI: -3.09 to -0.48) on 150 mg -The greatest effect on NRS pain intensity was found in subjects treated with 40 mg (- 1.57, 95% CI: 2.93 to -0.20) and with 150 mg (-1.34, 95% CI: - 2.51 to -0.17) -Similar effects were observed in sleep interference with 40 mg (- 0.49, 95% CI: -1.14 to 0.16) and with 150 mg (- 0.94, 95% CI: -1.78 to -0.09) -Reductions in McGill questionnaire pain intensity (measured on Visual Analogue Scale) were observed with 40mg (-17.3, 95% CI: -29.2 to -5.5) and with 150 mg -9.6 (95% CI: -19.0 to -0.1) -Improvement in PGIC and CGIC were also observed **Safety**: -Headache was the only treatment-related adverse event and cough was reported by 2 participants **Limitations**: -Limited baseline pain for study participants -Short washout period and overall study duration	-NRD.E1 emerges as a promising therapeutic approach in PDPN
Jain et al. (2022) (38)	Randomised, placebo- controlled, proof- of-concept trial	138 subjects with PDPN with mean age of 56.13 years, with mean BMI of 27.2 kg/m^2^ and mean time since diagnosis of DPN 795.6 days (91.9% of study participants with T2DM) -72 subjects treated with ISC 17536 -66 subjects in the control arm Exploratory subgroup with subjects with preserved small nerve fiber function function with moderate to severe pain (API> 5) -30 subjects treated with ISC 17536 -35 subjects in the control arm	**Efficacy**: -At week 4: reductions in 24- hour API score were 1.9 for the treatment arm and 1.7 for the control arm -Mean difference was not significant (- 0.26, 95% CI: 20.81-0.28) -In the exploratory population: significantly reduced pain score at week 4 on ISC 17536 (20.96, 95% CI: 21.68-20.24) -Significance at week 1 (20.82, 95% CI: 21.52- 20.12), week 2 (20.74, 95% CI: 21.45-20.03), week 3 (20.83 95% CI: 21.54- 20.12), and week 6 (21.37, 95% CI: 22.10-20.64) **Safety**: -Mild-to- moderate adverse events were reported with a similar frequency in both groups (31.9% in the treatment arm and 37.9% in the control arm); the most common adverse events were gastrointestinal (diarrhoea, dyspepsia and abdominal distension) **Limitations**: -Short study duration -Mostly Indian and male population included	- ISC 17536 holds therapeutic potential for subjects with preserved small nerve fiber function

PDPN, painful diabetic peripheral neuropathy; HbA1c, glycated haemoglobin; DM, diabetes mellitus; MNSI, Michigan Neuropathy Screening Instrument; T1DM, type 1 diabetes mellitus; T2DM, type 2 diabetes mellitus; CI, confidence interval; TEAEs, treatment emergent adverse events; S1R, sigma-1 receptor; DPNP, diabetic peripheral neuropathic pain; BMI, body-mass index; ADPS, average daily pain scores; NPSI, neuropathic pain symptom inventory; PGIC, patient global impression of change; HR, hazard ratio; RR, relative risk; DPN, diabetic peripheral neuropathy; NRS, numerical rating scale; UENS , Utah Early Neuropathy Scale; BPI- SF , Brief Pain Inventory-Short Form; NTSS-6, Neuropathy Total Symptom Score-6; Norfolk QOL-DN, Norfolk Quality of Life Questionnaire-Diabetic Neuropathy; UTI, urinary tractinfection; SFN, small-fibre neuropathy; WDPS, worst daily pain; OR, odds ratio; CGIC, Clinician Global Impression of Change; API, average pain intensity; NS, non-significant.

Self-perceived improvement in pain was reported by 52.4% of participants on E-52862 compared with 43.4% of subjects in the control arm (p=0.716) based on Patient Global Impression of Change (PGIC) (15). Neuropathic pain symptom inventory (NPSI) total pain intensity score was also similar between both study arms (p=0.422) (15). The same held true for rescue medication need (12.9% on E-52862 vs. 10.3% on placebo, p=0.642) (15). E-52862 was safe: only mild dizziness, nausea, headache, and anxiety were most frequently reported (15).

A large-scale phase 2 to 3 adaptive, multicenter, double blind, and placebo- and pregabalin-controlled randomised clinical trial evaluated the clinical efficacy of HSK16149 in 725 subjects with diabetic peripheral neuropathic pain (DPNP) (21). Various doses of gamma-aminobutyric acid (GABA) analogue HSK16149 (40, 80, 120, or 160 mg) were assessed at 2 distinct study stages (21). Average daily pain scores (ADPS) was used for treatment efficacy analysis which is largely based on the outcomes of NPRS. In the full-set analysis, there was a mean reduction in ADPS up to 1.13 in the 80 mg and 160 mg group vs. reductions by 0.75 in the control group and by 1.02 in the pregabalin group at week 5 (21). Subsequently, mean reduction by up to 2.24 was reached in the 40 mg group vs. reductions by 1.23 in the control group and by 2.09 in the pregabalin group at week 5 (21). Significance was obtained for subjects on 40 mg and 80 mg HSK16149 in terms of reduced pain perception (both p<0.25) (21). In the subgroup of subjects with DPNP duration >1 year, significantly reduced ADPS was again seen: -2.38 in the 40 mg group (p<0.001) and -1.89 in the 80 mg group (p=0.01) vs. -1.25 in the control arm (21). The most pronounced benefit was found for elderly subjects (≥65 years), subjects with DPNP duration exceeding 1 year and those on 80 mg HSK16149 (21). The significant benefit begun from week 8 on, for the same groups (21): 57.3% and 32.0% on 40 mg (vs. 31.6% on placebo) and 51.4% and 36.3% on 80 mg (vs. 18.1% on placebo) achieving at least a 30% and at least 50% pain decrease, respectively (21). Alternative pain assessment scales were also utilised: significant reductions in average daily sleep interference score (ADSIS) (-1.73 and -1.73, respectively) and visual analogue scale (VAS) (-22.9 and -23.0, respectively) on 40 mg and 80 mg HSK16149 were seen (p<0.001 for both) (21). The significant effect on pain alleviation was not similar on 40 mg and 80 mg throughout the different assessment tools used: the benefit was observed on 40 mg according to short form of the McGill Pain Questionnaire (SF-MPQ) scores (-3.50, p=0.009) assessing pain by taking into consideration the words chosen for sensory, affective and total descriptors at a scale from 0 to 3 for every descriptor (22) and in the EQ-5D-5L (an instrument to evaluate health status on 5 different dimensions (23)) scores (p=0 .008), but not on 80 mg (p=0.10 and p*=*0.07, respectively) (21). The same held true for SF-MPQ and PGIC scores (21). The frequency of adverse events was 75.1%, with mild-to-moderate dizziness and somnolence being the commonest (21).

### Accumulating evidence on pilavapadin (LX9211)

In a double-blind, randomised, placebo-controlled, proof-of-concept study, LX9211 was investigated at 2 doses: 10 mg and 20 mg (24). LX9211 is an orally administered selective, potent inhibitor of adapter protein-2-associated kinase 1 (AAK1) (25, 26). Although LX9211 mainly acts by alleviating nociception in the spinal cord, it was carefully developed to allow penetration into central nervous system (26). LX9211 is also under investigation for other forms of neuropathic pain, notably post-herpetic neuropathic pain (26). Furthermore, extensive *in vitro* profiling data both in humans and in multiple experimental models (rat, mouse, cynomolgus monkey and dog) showed decreased probability for drug-drug interactions due to cytochrome P450 (CYP) inhibition (26). Both doses were efficacious in alleviating pain by up to 1.39 at week 6 (LSMD -0.67, 95% CI: -1.16 to -0.18, p=0.007 and LSMD -0.55, 95% CI -1.06 to -0.05, p=0.030, respectively) (24).The dose of 20 mg did not reach the prespecified level of significance (24). Treatment with 10 mg and 20 mg LX9211 resulted in significant pain improvement vs. control arm (p=0.014 and p=0.017 vs. placebo, respectively), least pain (p=0.015 and p=0.020 vs. placebo) and interference of pain with sleep (p=0.005 and p=0.002 vs. placebo) (24). The beneficial effect for pain right now was restricted to low-dose treatment group (p=0.005) (24). At week 6, there was improved NPSI score (p=0.008 on 10 mg LX9211 and p=0.064 on 20 mg LX9211) and improved burning pain perception (p=0.008 on 10 mg LX9211 and p=0.064 on 20 mg LX9211) (24). Reductions by 30% and 50% in ADPS from baseline to week 6 were more pronounced for 10 mg LX9211 (27.4% and 15.1%, respectively) vs. placebo (17.8% and 10.3%, respectively), without significant difference for the 20 mg LX9211 group (17.0% and 9.4%) (24). Nevertheless, no significant differences vs. controls were observed in the proportion of subjects achieving reductions by 30% and 50% in ADPS (24).

According to PGIC, overall health improvement was significantly more frequent on 10 mg treatment group vs. control arm (least squares (LS): -0.35, 95% CI: -0.67 to -0.03, p=0.031), but not for the 20 mg treatment group (LS: -0.15, 95% CI: - 0.48 to - 0.17, p=0.351) (24). PGIC is regarded a validated tool for chronic pain treatment efficiency perception (27).

Common adverse effects were of mild-to-moderate severity and included dizziness, nausea, headache, constipation, balance disorder, somnolence and vomiting, presenting with an incidence over 5% for either dose group (24). There was a dose-dependent trend for dizziness, nausea, constipation and vomiting (24). The majority of adverse events on LX9211 were mild (51.5%) or moderate (41.6%) (24). Most adverse events were observed during the first week of treatment and were then ameliorated with continued LX9211 treatment (24). Treatment discontinuation mostly attributed to dizziness, headache, nausea and fatigue, occurred within the 2 first weeks on 10 mg LX9211 and within 6 weeks on 20 mg LX9211 (24). Importantly, there was similar incidence of adverse events in all groups (21.2% in the low-dose LX9211 group, 19.6% in the high-dose LX9211 group and 14.4% in the control arm) (24).

The findings of a recent phase 2b, dose-ranging, randomised controlled, multi-centre trial (PROGRESS) on the effects of pilavapadin (LX9211) are now available (28). Following treatment arms were included: subjects treated with 10 mg or 20 mg, subjects treated with 20 mg and after 7 days with 10 mg for the remaining of the study period (28). Significantly decreased ADPS were seen on pilavapadin vs. placebo (28). The greatest decrease in ADPS was found on 10 mg (-0.42) and on combined treatment with 20/10 mg (-0.40) vs. the control arm (28). The 10 mg pilavapadin treatment was associated with greater decrease in daily sleep interference (-1.60 vs. -1.19) and in burning pain (-1.6 vs. -1.1) (28). A *post-hoc* analysis excluding the study arm treated with 20 mg pilavapadin confirmed the significant effect of pilavapadin, showing a consistent early separation from the control arm during the study period (28). Dizziness and nausea were the most frequent adverse events in line with the previous study (28).

Given these promising outcomes in pain reduction, as well as the overall favourable pharmacologic profile linked with minor adverse events and low possibility for drug-drug interactions, pilavapadin should now be evaluated in phase 3 studies. Certainly, the comparison of pilavapadin with current standard-of-care medications in mitigating neuropathic pain, such as gabapentinoids is required to establish its therapeutic potential (7, 8). The latter require special considerations for specific subgroups of subjects DPNP, such as diminished starting doses and cautious uptitration (7). This was not suggested in currently available evidence from clinical studies. The most important benefit of pilavapadin is its selective mechanism of action by directly targeting a major contributor to DPNP development, AAK1 beyond mere symptomatic pain relief promoted by currently available agents.

### Further therapeutic approaches

The efficacy of mirogabalin, a novel voltage-gated Ca^2+^channel α2δ ligand was addressed in a model-based meta-analysis (29). ADPS decreased by 8.86% (95% CI: 4.6-12.94%) on pregabalin and by 7.57% (95% CI: 1.1-14.3%) on mirogabalin (29). Among mirogabalin users, weight gain (relative risk [RR]:7.12, 95% CI: 2.78-18.25), followed by peripheral oedema (RR: 4.88, 95% CI: 2.35-10.11), somnolence (RR: 3.74, 95% CI: 2.57-5.43) and dizziness (RR: 3.59, 95% CI: 2.31-5.60) were the main untoward effects (29). There was an insignificant reduction in dropout rate for mirogabalin (12.1%) vs. pregabalin (17%) and placebo (15.1%) (29).

Michelson et al. (30) conducted a phase 2, 12-week, randomised, double-blind, placebo-controlled study: 142 subjects received ricolinostat and 140 subjects were in the control arm (30). Ricolinostat is a histone deacetylase (HDAC)6 inhibitor, which is being appreciated in neuropathic pain, granted that HDAC6 may promote nociception (31–33). All assessment tools used in the study showed no benefit for ricolinostat compared with placebo: differences in numerical rating scale (NRS)(p=0.38), Utah Early Neuropathy Scale (UENS)(p=0.56), SF-BPI (p=0.74), Neuropathy Total Symptom Score-6 (NTSS-6) (p=0.72) and Norfolk Quality of Life Questionnaire-Diabetic Neuropathy (Norfolk QOL-DN) (p=0.93) were insignificant (30). Adverse events included urinary tract infections (3.5%), nausea (2.1%) and diarrhoea (2.1%) (30). There was a decrease in neutrophils in 27% of subjects on ricolinostat and 19% of those on placebo (30). An individual in the control arm was severely affected with neutrophil count below 1.25 10^3^/mm^3^ (30).

Vixotrigine was investigated in phase 2, multicentre, placebo-controlled, double-blind, enriched-enrolment, randomised withdrawal study (CONVEY) (34). Vixotrigine is a broad spectrum voltage-gated sodium channels (Navs) inhibitor, targeting both peripheral and central voltage-gated sodium channel subtypes (35). The study encompassed a carefully structured protocol aimed at properly selecting subjects: there was a 3-week screening period with daily electronic entries from the subjects screened and also a 5-day wash-out for possible use of pain medication (34). This was frequently necessary, considering the common use of pain-relieving medication: anticonvulsants (45.9%), tricyclic antidepressants (12.3%), other antidepressants (11.5%), opioids/opiates (17.2%) or other drugs (13.1%), namely thioctic acid, capsaicin, cannabidiol, and lidocaine (34). In the open-label study, subjects received 350 mg vixotriginetwice daily for a 4-week period (34). Then, 50.9% of participants experienced a decrease in ADPS exceeding 30% of the baseline values, and so they advanced to the next stage: participants were randomised according to a 1:1:1 ratio to receive 200 mg vixotrigine, 350 mg vixotrigine or placebo for 12 weeks (34). During this phase, ADPS, worst daily pain (WDP), and sleep interference were reported on a daily basis (34). A significant decrease in ADPS was found only on 200 mg vs. control arm (mean difference -0.85, 95% CI: -1.71 to 0.00, p=0.050 from baseline to week 12 and mean difference, -0.85, 95% CI: -1.74 to 0.03, p=0.058 from randomisation to week 12) (34). The effect on the group treated with 350 mg was insignificant in both analyses (mean difference, -0.17, 95% CI: -1.01 to 0.68, p=0.70 and mean difference, -0.32, 95% CI: -1.19 to 0.55, p=0.47, respectively) (34). In subjects with diabetic small fibre neuropathy (SFN), there was a beneficial effect for those on 200 mg vs. control arm (mean difference, -0.94, 95% CI: -1.85 to -0.03), but not for those on 350 mg (mean difference, -0.25, 95% CI: -1.18 to 0.69) (34).

At week 12, a significant reduction in worst daily pain scale (WDPS) was noted on 200 mg vs. control group (mean difference-0.93, 95% CI: -1.85 to -0.02, p=0.046, but not on 350 mg (mean difference -0.25, 95% CI: -1.15 to 0.65, p=0.58) (34). Nevertheless, not all analyses reached the same conclusion regarding optimal dose: significant improvement in PGIC vs. controls was seen on 350 mg (48.8% vs. 30.0%; odds ratio [OR]: 2.60, 95% CI: 0.97-6.99, p=0.058), but not on 200 mg (34). Insignificant improvement was observed in sleep interference score, NPSI total score, NPSI sum score of burning and pressing, and mean BPI-SF interference score (34). The safety profile was slightly different between the two substudies: in the open-label study, headache and dizziness of mild-to-moderate severity were the most frequent adverse events (9.4% in each treatment arm); in the double-blind study, falls, nasopharyngitis, muscle spasm and urinary tract infections were mostly reported (≥5% of vixotrigine-treated participants) (34).

A similar design was implemented in the study by Tiecke et al. (36) addressing the possible benefits of NRD.E1 treatment. NRD135S.E1 is an orally administered, lipophilic, small-molecule agent for neuropathic pain with poorly understood mechanism of action (37). At baseline, 25 participants received pain-relieving medication, including antiepileptics, antidepressants and analgesics (non-steroidal anti-inflammatory drugs [NSAIDs], paracetamol and opioids) (36). Decreased mean of daily average NRS pain intensity was found on 10 mg (0.42, 95% CI: -1.50 to 0.66, p=0.438), on 40 mg (0.82, 95% CI: 0.07 to 1.58, p=0.034), and on 150 mg (0.66, 95% CI: -0.03 to 1.35, p=0.061), but the pre-specified value of p=0.016 required was not met (36). Placebo-corrected NRS reductions were 0.52 (95% CI: -1.76 to 0.71) on 10 mg, 1.46 (95% CI: 0.26-2.66) on 40 mg and 1.20 (95% CI: 0.10-2.29) on 150 mg (36). In the sub-population of modified intent to treat subjects who had confirmed moderate or severe pain, placebo-corrected reductions by 1.69 (95% CI: -3.29 to -0.09) on 10 mg, by 2.66 (95% CI: -4.15 to -1.18) on 40 mg and by 1.78 (95% CI: -3.09 to -0.48) on 150 mg were found, corresponding to standardised effect size (SES) of -0.84 (95% CI: -1.64 to -0.04), -1.40 (95% CI: -2.18 to -0.62), and -1.00 (95% CI: -1.74 to -0.27) (36).The number needed to treat (NNT) for 30% response was 8.10 (95% CI: 2.46, -6.30), 2.85 (95% CI: 1.58, 13.92), 4.20 (95% CI: 1.90 to -20.0), for 10 mg, 40 mg and 150 mg, respectively. The NNT for 50% response was 12.15 (95% CI: 3.00 to -5.94), 3.78 (95% CI: 1.88 to -301), and 4.20 (95% CI: 1.96to -31.2), respectively (36).

In the same study, participants on 40 mg and 150 mg experienced the greatest reduction in NRS pain intensity (-1.57, 95% CI: 2.93 to -0.20) (36). Similar results were noted in sleep interference with 40 mg (-0.49, 95% CI: -1.14 to 0.16) and with 150 mg (-0.94, 95% CI: -1.78 to -0.09) (36). The McGill questionnaire pain intensity confirmed the improvement: reductions with 40 mg (-17.3, 95% CI: -29.2 to -5.5) and with 150 mg (-9.6, 95% CI: -19.0 to -0.1) were noted (36). Self-reported pain perception was also improved, based on PGIC and Clinician Global Impression of Change (CGIC) (36). Headache was the only adverse event (36).

In a randomised, placebo-controlled, proof-of-concept trial, ISC 17536 was examined as an oral inhibitor of Transient Receptor Potential Ankyrin 1 (TRPA1) in PDPN (38). TRPA1 is the only channel found in mammals from the category of transient receptor potential ankyrin channels (39). The latter are involved in acute and chronic pain by processing a wide variety of noxious external stimuli and of endogenous cell damage (39). The study included 138 subjects with PDPN, almost exclusively with T2DM (91.9%): 72 subjects received ISC 17536 and 66 subjects were allocated to the control arm (38). Similar reductions in 24-hour average pain intensity (API) score were seen in both groups (1.9 for the treatment arm and 1.7 for the control arm) (38). There was no difference in all pain assessment tools (night-time API, worst pain intensity, sleep interference PGIC, CGIC and NPSI) (38). At week 4, significantly reduced pain score was found for subjects on ISC 17536 (20.96, 95% CI: 21.68 to 20.24) in the exploratory population with high baseline pain (38).The effect was significant for week 1 (20.82, 95% CI: 21.52-20.12), week 2 (20.74, 95% CI: 21.45-20.03), week 3 (20.83, 95% CI: 21.54-20.12) and week 6 (21.37, 95% CI: 22.10 to 20.64) (38). In this subgroup, the percentage of patients who achieved reduction in pain score >50% was significantly greater on ISC 17536 vs. placebo (p<0.025) (38). The commonest adverse events were gastrointestinal, such as diarrhoea, dyspepsia, dysgeusia and abdominal distension (38). Diarrhoea and dysgeusia were more frequent in the treatment arm vs. control arm (38). Pain, possibly attributed to ISC 17536 was reported by 2 participants and none in the control arm (38). Nevertheless, adverse events of ISC 17536 cannot be reliably evaluated based on this earliest study, granted that all adverse events occurred in 1 (1.4%) or 2 (2.8%) participants in the treatment arm and 0 (0%) to 3 (4.5%) participants in the control arm (38). Transient moderate adverse events included: aspartate aminotransferase elevation, dyspepsia, emesis, abdominal distension and hyperchlorydria (38).

## Experimental agents in PDPN

DDD-028 was one of the major non-opioid agents with neuroprotective properties recently introduced for PDPN at doses of 1, 10 and 25 mg/kg (40). DDD-028 exerts its neuroprotective actions by suppressing both astrogliosis and axonal damage mediated through α7 nicotinic acetylcholine receptor (α7nAChR) (40). DPN was assessed based on mechanical allodynia through electronic von Frey test (40). Mechanical hyperalgesia was assessed through Paw Pressure test (40). The agent was compared with pregabalin (40). Pain relief was documented even with the lowest dose, but it had a short duration (30 minutes with 1 mg/kg and 90 minutes with 25 mg/kg) (40). In acute pain (hot plate and tail flick test assay), the benefit was not accomplished with 1, 3 and 10 mg/kg DDD-028 in contrast to 3 mg/kg morphine (40) (**Table 2**).

**Table 2:** Experimental studies on emerging therapeutic agents in PDPN.

Study	Study design and experimental model	Results	Conclusions
Micheli et al. (2023) (40)	10 rats with STZ- induced DPN treated with DDD-028 and pregabalin	-Rats treated with 1 mg/kg DDD-028: pain relief after 30 minutes lasting for 30 minutes -Rats treated with 25 mg/kg DDD-028 experienced pain relief for 90 minutes -Hot plate and tail flick test: rats on DDD-028 experienced no pain relief in contrast to those on morphine	-Experimental data points to therapeutic potential of DDD-028 in PDPN
Kuo et al. (2024) 41	-STZ-induced diabetic rats treated with J- 2156 -STZ-induced diabetic rats treated with gabapentin -STZ-induced diabetic rats treated with morphine -STZ-induced diabetic rats in the control arm	-Phase 1: J-2156 significantly alleviated hindpaw allodynia, reaching a maximal effect at 0.75 h with 20 mg/kg and at 1.5 h with 30 mg/kg vs. pregabalin after 1 h and vs. morphine after 1 h -Only 30 mg/kg J- 2156 significantly reduced allodynia vs. control arm (ΔPWT AUC10.2±0.8 g.h vs. 0.5±0.2 g h) and was comparable with 1 mg/kg morphine (13.8±1.9 g h) (p>0.05) -10 mg/kg J-2156 and 20 mg/kg J-2156: no benefit vs. controls -Phase 2: J-2156 administration had a shorter duration (1.5 h) compared to phase 1 - 30 mg/kg J-2156: 2-fold reduction of allodynia vs. the effect in phase 1 (4.7±1.3 g.h vs. 10.2±0.8 g h, p<0.05), comparable to 1 mg/kg morphine (13.8±1.9 g h, p>0.05) -10 mg/kg J-2156 and 20 mg/kg J-2156: no benefit vs. controls -Morphine: significant effect (13.8±1.9 g.h, p<0.05) and in phase 2 showed reduced effect (3.9±1.9) g h, p<0.05) -Pregabalin: similar effect (11.6±1.6 g.h, p>0.05) and in phase 2 showed similar effect (10.9±3.4 g.h, p>0.05) -All three agents had an action duration >3h	- J-2156, a SST4 receptor agonist holds therapeutic potential in PDPN
Peng et al. (2024) (44)	STZ-induced n Sprague-Dawley rats with PDN treated with PW507, gabapentin or vehicle (distilled water)	-At 1.5 h following PW507 administration: non- significant increase in PWT (p=0.44) -Gabapentin: significant alleviation of mechanical allodynia (p<0.001) -PW507: reduction of thermal hyperalgesia by 50% at 1 h and 2 h vs. controls (p<0.01) -Gabapentin: significantly reduced thermal hyperalgesia (p<0.01) -In the chronic study: on both day 21 (42.60±1.50 g vs. 31.28±0.78 g, p<0.0001) and on day 28 (40.79±0.98 g vs. 30.50±0.56 g, p<0.0001), PW507 significantly improved PWT vs. controls -On both day 21 (12.14±0.71 s vs. 9.40±0.71 s, p=0.03) and on day 28 (14.44±1.07 s vs. 8.24±0.79 s, p<0.0001), PW507 significantly improved PWL vs. controls	-PW507 treatment may be beneficial in PDPN

STZ, streptozocin; DPN, diabetic peripheral neuropathy; AUC, area under the curve; PDN, painful diabetic neuropathy; SEM, standard error of the mean; SST4, somatostatin receptor 4; PWT, paw withdrawal threshold; PWL, paw withdrawal latency.

J-2156, a somatostatin receptor 4 (SST4) receptor agonist was investigated at various doses (10, 20 and 30 mg/kg) and was directly compared not only with 100 mg/kg gabapentin, but also with 1 mg/kg morphine along with the control arm (41). J-2156 was introduced as a receptor selective peptidomimetic compound due to its capacity in ameliorating somatostatin release from capsaicin-sensitive afferent neurons (42). Researchers focused on a comprehensive assessment of PDPN, both in phase 1 which is regarded as morphine sensitive and in phase 2 which is considered partly morphine resistant (41).

In phase 1, J-2156 administration resulted in a significant effect on hindpaw allodynia reaching a maximal effect at 0.75 h with 20 mg/kg and 1.5 h with 30 mg/kg vs. pregabalin after 1 h and morphine after 1 h (41). Only 30 mg/kg J-2156 significantly alleviated allodynia vs. control arm (mean duration of allodynia alleviating effects, Δpaw withdrawal threshold [PWT] area under the curve [AUC]10.2 ± 0.8 g h vs. (0.5 ± 0.2 g h) and the effect was comparable to 1 mg/kg morphine (13.8 ± 1.9 g h) (41). No significant improvements were seen with 10 mg/kg J-2156 and 20 mg/kg J-2156 (41). In phase 2, the pharmacokinetic profile of J-2156 was different, as observed in the shorter duration (1.5 h) compared with phase 1 (41). The effect of 30 mg/kg J-2156 in alleviating allodynia was 2-fold reduced vs. the effect in phase 1(4.7 ± 1.3 gh vs. 10.2 ± 0.8 g h, p<0.05) (41). In line with phase 1, 10 mg/kg J-2156 and 20 mg/kg J-2156 (1.4 ± 0.5 and 1.2 ± 0.7 g h) showed no benefit vs. control arm (41).

RAP-103 was identified as a suitable agent for PDPN, as a complete reversal of mechanical and thermal hypersensitivity was assessed (43). Various doses of RAP-103 were found effective in improving mechanical allodynia (0.5 mg: p<0.001; 0.1 mg: p<0.05; 0.02 mg: p<0.05), with the exception of 0.004 mg (43). Similar results were obtained for cold allodynia (0.5 mg: p<0.01; 0.1 mg: p<0.05; 0.02 mg: p<0.05) (43). In sciatic nerves of rats with PDPN, RAP-103 significantly reduced interleukin (IL)-1β (p<0.001), tumour necrosis factor (TNF)-α (p<0.05) and chemokine (C-C motif) ligand 1 (CCL)3 (p<0.05) vs. age-matched non-DPN control rats (43). Similar outcomes were found in spinal cord (TNF-α: p<0.01; C-C chemokine receptor (CCR)5: p<0.01) (43).

PW507 (another S1R antagonist) was tested in a STZ-induced PDPN model (44). Thermal hyperalgesia was effectively reduced by 50% at 1 h and 2 h following PW507 administration vs. controls (p<0.01) and similar outcomes were observed with gabapentin (p<0.01) (44). Repeated PW507 administration resulted in alleviation of both mechanical (increased PWT) and thermal hyperalgesia (increased paw withdrawal latency [PWL]) (44).

Liu et al. (45) et al. studied BH177, a positive allosteric modulator of gamma-aminobutyric acid B receptor (GABA_B_) receptors. BH177 increased availability of GABA_B_ receptors in the dorsal horn of the rat spinal cord in a STZ-induced PDPN (45). CGP4638, a GABA_B_ receptor antagonist administration prevented this increase (45). Subsequent increases in PWT and PWL was achieved by suppressing the protein kinase C (PKC)/calcium/calmodulin-dependent protein kinase II (CaMKII)/extracellular signal-regulated kinase (ERK)1/2/cyclic adenosine monophosphate (cAMP) response element-binding protein (CREB) signaling pathway and by promoting activation of GABA_B_ receptors (45).

It has also been attempted to target chemokines and other inflammatory mediators (46–49). Cenicriviroc (a dual CCR2/CCR5 antagonist) was examined in Swiss albino mice with streptozocin (STZ)-induced DPN (46). It effectively ameliorated mechanical (p<0.001) and thermal hypersensitivity in male mice at 2 h, 4 h and 6 h (46). Opioid receptor expression was significantly decreased in male mice, without any effect on female mice. Opioid Receptor Mu (Oprm)1 (p<0.05) and Opioid Receptor Delta (Oprd)1 (p<0.01) were mostly affected (46). The 5 mg/kg dose accomplished a more pronounced effect in male vs. female mice (46).

Ladarixin acts in the CXC motif chemokine ligand 8 (CXCL8)-CXC motif chemokine receptor 1/2 (CXCR1/2) axis (47). It reduced mechanical allodynia vs. controls at all timepoints (1–4 weeks, 5–8 weeks and 5 weeks) (47).

Administration of AMD3100 (a CXCR4 antagonist) resulted in complete reversal of decreased PWT and PWL at week 2 (48). In addition, increased expression of toll-like receptor (TLR)9 is gaining attention (49). In rats, lentivirus-mediated TLR9 knockdown delivered to spinal cord significantly reduced mechanical withdrawal thresholds (MWT) and PWL (49).

Moreover, transient receptor potential vanilloid 1 (TRPV1) and GPCRs have been explored as potential therapeutic targets (50–53). The former are largely involved in neuropathic pain (54–57). Therapeutic silencing of P2X purinoceptor 7 (P2X7) has been shown to improve MWT and thermal withdrawal latency (TWL), as well as to reduce TRPV1 expression (50).

Xie et al. (51) studied G protein-coupled receptor 177 (GPR177). This is an orphan G protein-coupled receptor (GPCR) (57). It promotes PDPN development through TRPV1. This is mediated by WNT5a secretion from A-fibre dorsal root ganglion (DRG) neurons into cerebrospinal fluid (51). WNT5a targeted the extracellular S5-S6 loop of TRPV1 (51). The use of a peptide targeting this WNT5a/TRPV1 interaction was effective in suppressing PDPN (51).

Vascular endothelial growth factor (VEGF)-A promotes thermal hyperalgesia (52). In a mouse model, VEGF-A_165_ b (an endogenous inhibitor of VEGF) prevented the development of neuropathic pain (53).

Moreover, a local therapy was examined in Wistar rats and mini pigs with STZ-induced PDPN (58). Significantly reduced PWL was found for rats with PDPN (5.5 ± 1.1s, p<0.01), and this effect was reversed following resiniferatoxin cream application (58). Progressively decreasing PWT were seen (week 1: 25.4 ± 9.93%; week 5: 5.8 ± 0.76%, p<0.05).These were gradually restored following resiniferatoxin application (week 1: 22.4 ± 10.8%; week 5: 5.51 + 5.6%, p<0.05) (58). Significantly increased paw skin calcitonin gene-related peptide (CGRP) release vs. controls in rats with PDPN (531 ± 26 pg/ml/mg vs. 232 ± 8 pg/ml/mg, p<0.01) was found. Accordingly, it was hypothesised that resiniferatoxin directly targeted CGRP release (58). This was further substantiated, as CGRP release was promoted by capsaicin administration and was significantly decreased following resiniferatoxin cream application (p<0.01) (58). As a second model, mini-pigs were used. In these, resiniferatoxin cream reduced pain vs. untreated animals (p<0.05) (58). Following resiniferatoxin cream application, 1 μM capsaicin-enhanced CGRP release was significantly lower vs. controls (1953 ± 139 pg/ml/mg, vs. 4817 ± 255 pg/ml/mg, p<0.01) (58). TRPV1 expression was significantly reduced after treatment with resiniferatoxin cream (0.067 ± 0.01 vs. 0.63 ± 0.012, p<0.011) (58). In skin biopsies from the body regions of mini-pigs treated with the cream, TRPV1 labelling correlated with neuropathic symptoms and treatment efficacy (58).

Potential epigenetic targets have also been explored (59–62). Zhang et al. (59) et al. studied micro ribonucleic acid (miR)-497 in DRG from rats with DPNP. Increases in PWT, PWL and motor nerve conduction velocity (MNCV), reaching control levels, were observed following administration of miR-497 (59). The latter is involved in promoting degradation of nuclear factor erythroid 2-related factor 2 (NRF2) and glucose-6-phosphate dehydrogenase (G6PD) (59). Previous research has demonstrated positive effects on PDPN by targeting aquaporin (AQP)4 with the use of natural products (60, 61). Treatment with β-hydroxybutyrate (BHB) (an inhibitor of class I histone deacetylases) significantly improved PWT (62).

Mitochondrial dysfunction as a potential target has been explored as well (63, 64). In DRG neurons from a PDPN model, Yuan et al. (63) showed that poly (adenosine diphosphate [ADP] –ribose) polymerase (PARP)1 inhibition improved mitophagy influx and mitochondrial membrane potential (MMP). The involvement of poly (adenosine diphosphate [ADP]-ribose) polymerase (PARP) in DPN is well-established for years and is gaining increasing attention in experimental settings (65, 66).George et al. (64) showed that mitochondrial calcium uniporter deletion (which effectively prevents calcium entry into mitochondria) attenuated axonal degeneration and mechanical allodynia.

## The therapeutic potential of antidiabetic medication in PDPN

Zhang et al. (67) assessed the role of the glucagon-like peptide-1 receptor agonist (GLP-1RA) liraglutide in ameliorating PDPN. They used a STZ-induced T1DM model of male Sprague-Dawley rats, as well as the microglial cell line BV2 (67). At 4 weeks following STZ injection, mechanical (p=0.003) and thermal pain thresholds (p=0.032) were significantly increased compared with the control arm (67). Intra-cerebroventricular injection of liraglutide reduced mechanical (p=0.0005) and thermal pain (p=0.0003) thresholds (67) (**Table 3**). It also reduced inflammation, as evidenced by significantly reduced TNF-α, IL-6, and IL-1β in the brain of rats with PDPN (67). Nevertheless, in BV2 microglia, liraglutide at 50 µM and 100 µM resulted in decreased cell viability (67). Exposure of microglia to lipopolysaccharide (LPS) or high glucose increased messenger ribonucleic acid (mRNA) and concentrations of TNF-α, IL-6 and IL-1β in BV2 microglia, and liraglutide reversed these changes (67).

**Table 3:** Experimental studies on the potential utility of antidiabetic agents in PDPN.

Study	Study design and experimental model	Results	Conclusions
Zhang et al. (2022) (67)	-Male Sprague- Dawley rats with STZ-induced T1DM on liraglutide -Murine microglial line BV2	-At 1 h and 2 h following intra- cerebroventricular injection of liraglutide significantly increased mechanical (p=0.0005) and thermal pain (p=0.0003) thresholds -GLP-1R expression was confirmed in cortex of rats with PDPN and in BV2 microglia pointing to potential effects of GLP-1RAs -Significantly reduced mRNA levels of TNF-α, IL-6 and IL-1β in rat brain with PDPN, indicative of inflammation alleviation were found following liraglutide treatment -LPS administration resulted in the upregulation of 1147 genes and downregulation of 1468 genes; liraglutide resulted in the upregulation of 921 genes and downregulation of 1212 genes; 504 (13.5%) genes were significantly up-regulated by LPS and downregulated following liraglutide -NOD-like receptor pathway activity was enhanced in BV2 microglia treated with LPS and suppressed following coadministration of liraglutide and LPS	- Intra- cerebroventricular administration of liraglutide improves neuropathic pain
Lee et al. (2024) (68)	-Male Wistar rats with STZ-induced PDPN: -Rats treated with 1.44 ml/kg semaglutide -Rats treated with 2.88 ml/kg semaglutide -Control arm	-With semaglutide, MPWL increased by 25% and TPWL by 18% -In rats with PDPN, significantly increased AGEs were found compared with the control arm (115±5.5 µg/mL vs. 50±4.5 µg/mL, p<0.05); AGEs were significantly decreased following treatment with semaglutide (p<0.05) -TNF-α, IL-1β and IL-6 expression in spinal dorsal horn was significantly reduced with semaglutide -Significantly increased expression of IBA-1 and GFAP was found in the spinal dorsal horn and subsequently reduced with semaglutide -Increased TNF-α and IL- 1β expression was found in rats with PDPN and subsequently reduced with semaglutide	- Semaglutide is an emerging therapeutic option in PDPN
Santos et al. (2022) (69)	-ZDF rats treated with 100 mg/kg pioglitazone - db/db mice treated with 100 mg/kg pioglitazone	-Pioglitazone significantly reduced heat hypersensitivity 60 minutes after intra-peritoneal injection in both male (p=0.0023) and female ZDF rats (p=0.0009); similar results were shown for mechanical hypersensitivity for male (p=0.0001) and female mice (p=0.0054) -In db/db mice, pioglitazone significantly reduced heat hypersensitivity for female (p=0.0036) and male mice (p<0.0001) -6-week treatment with did not alleviate heat hypersensitivity in male or female rats	-Pioglitazone had a short-term beneficial effect on PDPN in mice

PDPN, painful diabetic peripheral neuropathy; T1DM, type 1 diabetes mellitus; STZ, streptozocin; DNP, diabetic neuropathic pain; DPA-714, N-diethyl-2-[4-(2-fluoroethoxy)phenyl]-5,7-dimethylpyrazolo[1,5-a]pyrimidine-3-acetamide; GLP-1RAs, glucagon-like peptide-1 receptor agonists; GLP-1R, glucagon-like peptide-1 receptor; PDPN, painful diabetic peripheral neuropathy; TNF, tumour necrosis factor; IL, interleukin; LPS, lipopolysaccharide; NOD, nucleotide-binding oligomerization domain; NLRP3, Nucleotide- Binding Domain, Leucine-Rich-Containing Family, Pyrin Domain-Containing-3; PWL, paw withdrawal latency; MPWL, mechanical paw withdrawal latency; TPWL, thermal paw withdrawal latency; AGE, advanced glycation end-products; IBA-1, ionised calcium-binding adaptor molecule 1; GFAP, glial fibrillary acidic protein; ZDF, zucker diabetic fatty; CCK8, cell counting kit-8.

In another work using male Wistar rats (68), PDPN was associated with reduced mechanical paw withdrawal and thermal paw withdrawal threshold latencies, as well as increased advanced glycation end-products (AGEs). Semaglutide increased mechanical paw withdrawal and thermal paw withdrawal threshold latencies by 25% and 18%, respectively (68). AGEs were significantly reduced by semaglutide (p<0.05) (68). Microglial hypertrophy and somatic and dendritic hypertrophy of astrocytes were restored with semaglutide (68). Glycated haemoglobin (HbA_1c_), very low-density lipoprotein cholesterol (VLDLc) and low-density lipoprotein cholesterol (LDLc) were significantly reduced with semaglutide, as well (68).

Pioglitazone was also examined in PDPN (69). Zucker diabetic fatty (ZDF) rats, homozygous for the loss-of-function “fatty” mutation in the leptin receptor, developed significantly increased mechanical hypersensitivity at 15–16 weeks of age vs. male and female ZL rats, acting as heterozygous (fa/+) genetic controls (p=0.01) (69). Treatment with pioglitazone (100 mg/kg) significantly decreased heat hypersensitivity 60 minutes after intra-peritoneal injection, in both male (p=0.0023) and female ZDF rats (p=0.0009) (69). Similar results were shown in mechanical hypersensitivity for male (p=0.0001) and female mice (p=0.0054), without any difference between male and female cohorts (69). In this work, db/db mice with a similar genetic background were included as an equivalent mouse model (69). In db/db mice, pioglitazone significantly reduced heat hypersensitivity in female (p=0.0036) and male mice (p<0.0001) (69).

Sodium-glucose cotransporter-2 inhibitors (SGLT-2is) may also hold potential (70). Daily oral empagliflozin has been shown to reduce pain hypersensitivity (70), but more experience is needed.

Previous research has also yielded favourable outcomes for various antidiabetic agents. Metformin has shown ameliorating potential for hyperalgesia and allodynia in STZ-induced PDPN in a rat model (71). This effect may be attributed to the activation of 5' adenosine monophosphate-activated protein kinase (AMPK), holding a functional link with TRPA1 (72). Another study in diabetic rats showed that the analgesic effects of metformin could be mediated by diminished nuclear factor kappa-light-chain-enhancer of activated B cells (NF-κB) expression along with AMPK activation in the lumbar DRG (73). Reduced synapses in the spinal dorsal horn have been proposed as a further mechanism (74).

The emerging value of GLP-1RAs in PDPN has been concluded in various works. Glucagon-like peptide-1 receptor (GLP-1R) activation in microglia from the spinal dorsal horn was linked with reduction of pain hypersensitivity by acting in part though β-endorphin signalling (75). The same pathway was stimulated by the non-peptide GLP-1RA WB4–24 (76). The significance of GLP-1R activation was confirmed in subsequent studies (77, 78). Dipeptidyl-peptidase-4 inhibitors (DPP-4is) have gained limited interest for PDPN (79, 80). Inflammation-driven DPP-4 expression was significantly enhanced promoting hyperalgesia development (81). This effect was suppressed following administration of DPP-4is, the tripeptide isoleucin-prolin-isoleucin and vildagliptin (81). This effect was partly mediated by opioid receptors (81). In a sciatic partial transaction Wistar rat model, mechanical allodynia, thermal hyperalgesia and tail-flick response were improved following teneligliptin administration (82).

Although less evidence is available regarding the potential benefit of pioglitazone, similar encouraging outcomes pointing to reduced hyperalgesia and improved nociception have been demonstrated (83).

## Discussion

This review has summarised clinical and experimental evidence on new and emerging pharmacological agents for PDPN. Main agents in clinical studies include E-52862 (a S1R antagonist (15)), HSK16149 (a GABA analogue (21)), LX9211/ pilavapadin (an inhibitor of AAK1 (24, 28)), micorgabalin (a voltage-gated Ca^2+^ channel α2δ ligand (29)), ricolinostat (a HDAC6 inhibitor (30)),vixotrigine (a non-specific Navs inhibitor (34)), NRD135S.E1 (36) and ISC 1536 (a TRPA1 inhibitor (38)). Preliminary outcomes for E-52862, ricolinostat or ISC 17536 were not encouraging (15, 30, 38). Other agents were considered beneficial, but failed to meet study endpoints, such as NRD135S.E1 (36). The most promising agents were HSK16149, LX9211/ pilavapadin, microgabalin and vixotrigine (21, 24, 28, 29, 34). These agents merit further investigation in large clinical trials, as existing evidence is largely based on single clinical studies with the notable exception of LX9211/ pilavapadin. Some agents were particularly beneficial for carefully selected individuals with PDPN: e.g. elderly subjects with established PDPN for longer than 1 year on HSK16149 (21). The benefit for elderly subjects should be further explored. Indeed, titration of existing medications to this subgroup of individuals is extremely challenging due to comorbidities (84, 85). ISC 17536 treatment failed to show any pronounced improvement in the general study population, but was favourable for subjects with high baseline pain intensity (38). This outcome is of great interest, granted that the use of opioids is strongly discouraged in PDPN (7, 86). A recent neuroimaging study suggested detrimental effects due to impaired dopaminergic signalling (87).

The safety profile of the new agents was highly heterogeneous, but most adverse events were of mild-to-moderate severity: nausea, constipation, vomiting, and diarrhoea (15, 24, 30, 38). Headache was also reported (15, 24). Less common adverse events included urinary tract infections (30, 34). Some agents showed no consistent adverse events in various sub-studies, thus pointing to the need for additional safety studies (34).

Overall, there was great heterogeneity in terms of populations (ranging from merely 88 individuals (36) to 752 individuals (21)) or duration (between 4 weeks (15) and 13 weeks (24)). Some studies included both T1DM and T2DM subjects (15, 24). In the majority of the included studies, a comparison with placebo was present, whereas some trials focused on direct comparisons with other commonly used analgesic agents, such as pregabalin (21, 29). Diverse pain assessment tools were used, the most common being ADPS (21, 29, 34, 38), NPSI (15, 24, 34, 38), PGIC (21, 24, 34, 36, 38) and sleep interference tools (21, 24, 28, 34, 36, 38), such as ADSIS (21).

Experimental works have included a wide variety of novel agents: PW507 (a S1R antagonist (44)), BH177 (a GABA_B_ agonist (45)) and BHB (a HDAC inhibitor (62)). SST4 inhibition through J-2156 posed another potential mechanism (41). Selective chemokine suppression, e.g. cenicriviroc (a dual CCR2/CCR5 (46)) or ladarixin (an inhibitor of CXCR1/2 (47)) hold important potential. Mitochondrial involvement is also emerging, as shown by the beneficial effects of PARP1 inhibition by PJ34 (63). Administration of VEGF inhibitor axitinib or GPR40 agonist GW9508 may also prove useful (52). Furthermore, resiniferatoxin has been suggested as an effective local treatment (58). Certainly, these novel agents unravel emerging pathophysiological pathways, but only a few of them may prove to be clinically useful.

Experimental evidence on antidiabetic medication is very interesting but extremely limited, with liraglutide and semaglutide holding the greatest potential (67, 68). A single study reported beneficial effects of liraglutide through an unconventional intra-cerebroventricular delivery, possibly mediated through nucleotide-binding oligomerization domain, leucine-rich repeat (LRR)-, and pyrin domain (PYD)-containing protein 3 (NLRP3) suppression (67). Next-generation GLP-1RAs may hold even greater therapeutic potential in PDPN. Their role in PDPN management merits further investigation in randomised controlled trials (88). Behavioural assays included mostly von Frey filaments which certainly pose one of the most frequently used and established pain assessment tools (89, 90). Various other antidiabetic agents, including pioglitazone or SGLT-2is, have been evaluated in a few experimental studies showing promising effects. However, to date clinical evidence would be of particular significance to determine whether users of specific agents experience indirect positive effects on PDPN severity as well. Large-scale observational studies could confirm benefit observed in experimental settings.

Several clinical trials evaluating novel agents in the management of PDPN are ongoing on a global scale. In the latest years, multiple novel therapeutic agents have been introduced in clinical studies. Candidate agents for PDPN include: CNTX-6016 (NCT04857957, phase 1b); MT-8554 (NCT05123196, Randomized, Double-Blind, Placebo-Controlled, Exploratory Study); VX-548/suzetrigine (vs. pregabalin in a phase 2 study, NCT05660538 and in a phase 3 study, NCT06696443 and vs. placebo or pregabalin in a phase 3 study, NCT06628908); Engensis (NCT04873232, Phase 3, previously assessed in NCT02427464 with promising outcomes (91)); eptinezumab, a CGRP monoclonal antibody (NCT05937152, phase 2); LY3556050 (NCT06074562, phase 2); AJH-2947 (NCT06155487, phase 1); the novel combination therapy of cagrilintide B and semaglutide I (NCT06797869); BAY1817080 (vs. pregabalin in a phase 2a/b study), VX-993 (NCT06619860, vs. placebo or pregabalin in a phase 2 study); Adezunap (AP707) as a tetrahydrocannabinol (THC)-focused nano endocannabinoid system modulator (NCT06072573, NCT06071975); BAY2395840 as a bradykinin B1 receptor (NCT05219812, phase 2); LY3848575 (NCT06568042, phase 2).

The strength of this review is the comprehensive assessment of preclinical and clinical evidence. Limitations in evidence may be summarised as follows. First, clinical trials are heterogeneous in design, not allowing direct comparisons: some included several stages and various study endpoints, and there was a wide spectrum of pain assessment scales. Noteworthy limitations in study design were also present. First, study duration was short, resulting in absence of long-term efficacy and safety data. Secondly, most data was from a single ethnic background (mostly of white origin), thus not allowing generalisation of evidence for other populations. Great heterogeneity is also evident in experimental works: researchers utilised a variety of animal models, not directly comparable with each other and different forms of medications (e.g. local cream application). Moreover, the exact effect of antidiabetic medication on PDPN development and progression has been only marginally studied. Finally, there are no large randomised controlled trials to provide more robust evidence.

## Conclusion

PDPN poses a major challenge for subjects with both T1DM and T2DM. Current treatment approach is restricted to symptomatic pain relief with various degrees of response among affected individuals. Clinical trials have confirmed the great potential of several novel agents, LX9211/ pilavapadin being the most notable example. Ongoing experimental research is pointing to novel mediators and additional pathways with potential clinical implications, thus promoting the development of agents selectively targeting these pathways. Furthermore, early experimental data points to potential encouraging effects of several antidiabetic agents, e.g. GLP-1RAs. Large clinical trials are required. These should validate promising outcomes and examine their clinical utility.

## Glossary

AAK1: adapter protein-2-associated kinase 1
ADP: adenosine diphosphate
ADPS: average daily pain scores
ADSIS: average daily sleep interference score
AGEs: advanced glycation end-products
AMPK: 5' adenosine monophosphate-activated protein kinase
API: average pain intensity
AQP: aquaporin
AUC: area under the curve
BHB: β-hydroxybutyrate
CaMKII: calcium/calmodulin-dependent protein kinase II
cAMP: cyclic adenosine monophosphate
CCL: chemokine (C-C motif) ligand
CCR: C-C chemokine receptor
CGIC: Clinician Global Impression of Change
CGRP: calcitonin gene-related peptide
CI: confidence interval
CREB: cyclic adenosine monophosphate (cAMP) response element-binding protein
CXCL: CXC motif chemokine ligand
CXCR: CXC motif chemokine receptor
CYP: cytochrome P450
DM: diabetes mellitus
DPN: diabetic peripheral neuropathy
DPNP: diabetic peripheral neuropathic pain
DPP-4: dipeptidyl-peptidase-4
DPP-4is: dipeptidyl-peptidase-4 inhibitors
DRG: dorsal root ganglion
ERK: extracellular signal-regulated kinase
G6PD: glucose-6-phosphate dehydrogenase
GABA: gamma-aminobutyric acid
GABA_B_: gamma-aminobutyric acid B receptor
GLP-1R: glucagon-like peptide-1 receptor
GLP-1RA(s): glucagon-like peptide-1 receptor agonist(s)
GPCR(s): G protein-coupled receptor(s)
GPR177: G protein-coupled receptor 177
HbA_1c_: glycated haemoglobin
HDAC: histone deacetylase
IL: interleukin
LDLc: low-density lipoprotein cholesterol
LPS: lipopolysaccharide
LS: least squares
LSMD: least squares mean difference
miR: micro ribonucleic acid
MMP: mitochondrial membrane potential
MNCV: motor nerve conduction velocity
MNSI: Michigan Neuropathy Screening Instrument
mRNA: messenger ribonucleic acid
MWT: mechanical withdrawal threshold
Navs: voltage-gated sodium channels
NF-κB: nuclear factor kappa-light-chain-enhancer of activated B cells
NLRP3: nucleotide-binding oligomerization domain, leucine-rich repeat (LRR)-, and pyrin domain (PYD)-containing protein 3
NNT: number needed to treat
Norfolk QOL-DN: Norfolk Quality of Life Questionnaire-Diabetic Neuropathy
NPRS: numerical pain rating scale
NPSI: neuropathic pain symptom inventory
NRF2: nuclear factor erythroid 2-related factor 2
NRS: numerical rating scale
NSAIDs: non-steroidal anti-inflammatory drugs
NTSS-6: Neuropathy Total Symptom Score-6
Oprd: Opioid Receptor Delta
Oprm: Opioid Receptor Mu
P2X7: P2X purinoceptor 7
PARP: poly (adenosine diphosphate [ADP]-ribose) polymerase
PDPN: painful diabetic peripheral neuropathy
PGIC: Patient Global Impression of Change
PKC: protein kinase C
PWL: paw withdrawal latency
PWT: paw withdrawal threshold
RR: relative risk
S1R(s): sigma-1 receptor(s)
SES: standardised effect size
SF-BPI: Brief Pain Inventory-Short Form
SF-MPQ: short form of the McGill Pain Questionnaire
SFN: small-fibre neuropathy
SGLT-2is: sodium-glucose cotransporter-2 inhibitors
SST4: somatostatin receptor 4
STZ: streptozocin
T1DM: type 1 diabetes mellitus
T2DM: type 2 diabetes mellitus
THC: tetrahydrocannabinol
TLR: toll-like receptor
TNF: tumour necrosis factor
TRPV1: transient receptor potential vanilloid 1
TRPA1: transient receptor potential ankyrin 1
TWL: thermal withdrawal latency
UENS: Utah Early Neuropathy Scale
VAS: visual analogue scale
VEGF: vascular endothelial growth factor
VLDLc: very low-density lipoprotein cholesterol
WDP(S): worst daily pain (scale)
ZDF: Zucker diabetic fatty
7nAChR: α7 nicotinic acetylcholine receptor.
